# Association of Pulmonary Hypertension With Inflammatory Markers and Volume Status in Hemodialysis Patients of End-Stage Renal Disease

**DOI:** 10.7759/cureus.13635

**Published:** 2021-03-01

**Authors:** Satyendra K Sonkar, Mahboob Alam, Sharad Chandra, Gyanendra K Sonkar, Anil Gaikwad, Vivek Bhosale

**Affiliations:** 1 Medicine, King George's Medical University, Lucknow, IND; 2 Medicine, King George’s Medical University, Lucknow, IND; 3 Cardiology, King George’s Medical University, Lucknow, IND; 4 Biochemistry, King George's Medical University, Lucknow, IND; 5 Pharmacology, Central Drug Research Institute, Lucknow, IND; 6 Clinical and Experimental Medicine, Central Drug Research Institute, Lucknow, IND

**Keywords:** alpha-1-acid glycoprotein, ejection fraction, fluid overload, pro-b-type natriuretic peptide, volume status

## Abstract

Background and objectives

Pulmonary hypertension (PH) is an independent risk factor for increased mortality, especially in patients undergoing hemodialysis (HD), but the mechanism of its development is unknown. This study aimed at evaluating volume overload and inflammation as potential variables to cause its development in patients undergoing maintenance hemodialysis.

Materials and methods

This was an observational cross-sectional study conducted on patients undergoing hemodialysis at a tertiary hospital in northern India. Patients of end-stage renal disease, aged 18 years or more, on maintenance hemodialysis for over two months were included in the study. The patients were divided into two groups based on the presence or absence of PH, determined by measuring systolic pulmonary arterial pressure (SPAP). The severity of PH was defined as: mild (SPAP 35-45 mmHg), moderate (SPAP 46-55 mmHg), and severe (SPAP> 55mmHg). The two groups were evaluated for demographic variables, type of vascular access, biochemical parameters, and markers of inflammation and fluid overload. Data between the two groups were compared statistically.

Results

This study included a total of 82 patients showing the prevalence of PH to be 25.6% with a men-to-women ratio of 2:1. Out of 21 cases of PH, mild PH was found in seven (33.3%) cases, moderate in 14 (66.7%), and cases with severe PH were none. The two groups differed significantly in ejection fraction and markers of inflammation and volume status. Laboratory data associated with PH were alpha-1-acid glycoprotein (p<0.05) and pro-b-type natriuretic peptide (p <0.05).

Conclusion

The present study showed higher levels of inflammatory markers alpha-1-acid glycoprotein and pro-b-type natriuretic peptide and lower levels of ejection fraction in patients undergoing HD, indicating a significant association with PH.

## Introduction

Despite, advances in the field of medicine, morbidity and mortality remain very high in patients with chronic kidney disease (CKD)/end-stage renal disease (ESRD). Cardiovascular complications are the leading contributing factors for high morbidity and mortality in this group of patients. Evidence suggests that even mild to moderate renal impairment increases the risk of cardiovascular disorders [1]. Among cardiovascular complications of ESRD, pulmonary hypertension (PH) is an important entity which itself is an independent risk factor for increased mortality especially in patients undergoing hemodialysis (HD) [2, 3]. Patients with CKD and PH are at increased risk of heart failure [2]. The prevalence of PH in patients undergoing HD has been reported to be as high as 50% in previous studies [4, 5]. Though multiple studies have tried to look into the factors which lead to the development of PH in patients undergoing HD, it largely remains unknown. Variables like age of patients, duration of HD, type of arteriovenous access, endothelial dysfunction, bone mineral disorders leading to vascular calcifications, fluid overload, increased oxidative stress and inflammation, systolic and diastolic dysfunction have been suggested as factors that lead to the development of PH in patients on HD. Moreover, inflammation and fluid overload in a patient with CKD interact and augment each other [6].

However, these observations are based mostly on retrospective studies, and inflammatory markers have been evaluated in very few studies. Therefore, the present study is aimed to evaluate the role of inflammation in the development of PH and its interrelationships with fluid overload in patients with ESRD on maintenance HD.

## Materials and methods

This was an observational cross-sectional study conducted in the dialysis unit of the department of medicine at a tertiary care center in northern India. A sample size of 81 patients was calculated from the following equation:

$SS = z^{2}\times \frac{p(1-p)}{d^{2}}$

Where,

z = standard normal variate (at 5% type I error [p<0.05] it is 1.96)

p = expected prevalence depending upon previous studies

d = margin of error

taking, p=30% and d=10%, sample size (SS) comes out to be 81.

Patients of ESRD, age greater than 18 years, on maintenance HD for over two months and who gave written informed consent were included in the study. Patients with the presence of ventricular dyskinesia or hemodynamically significant valvular disease, alcohol dependence, psychiatric disorders, chronic liver disease, malignant neoplasm, acute infectious diseases, other chronic inflammatory diseases, chronic pulmonary diseases and patients having a positive human immunodeficiency virus serology were excluded. The study was approved by the Institutional Ethics Committee. The procedure was carried out in accordance with the Declaration of Helsinki and the International Council for Harmonization-Good Clinical Practice (ICH-GCP).

The participants were divided into two groups on the basis of the absence or presence of pulmonary arterial hypertension. The clinical and laboratory findings were compared between these two groups. Demographic and clinical data (comorbidities, type and location of vascular access, blood pressure) were recorded. Blood samples were collected for testing complete blood count, sodium (Na+) / potassium (K+), urea /creatinine, liver function test, total protein, albumin, C-reactive protein (CRP), pro-b-type natriuretic peptide (Pro-BNP), alpha-1-acid glycoprotein (A1AG). Commercially available enzyme-linked immunosorbent assay (ELISA) kits were used to measure A1AG. The plasma and serum were centrifuged and frozen at -70 °C until further laboratory analysis.

The participants were dialyzed with a polysulfone dialyzer for 4 h, twice a week, in controlled ultrafiltration HD machines. Dialysate and blood flux were prescribed to reach a Kt/V target of 1.4. Anticoagulation was performed by heparin bolus during the HD procedure. The blood pressure monitoring was measured before dialysis and was considered the mean of measures immediately before each ten dialysis sections. Pre-HD and post-HD weights were recorded. 2D echocardiography (M mode) was done the next day after HD (in a relative euvolemic state) according to a previously standardized technique using an MyLab™Seven device ( Esaote S.p.A, Genoa, Italy) attached to a multi frequential 2.5 MHz to 3.5 MHz transducers. The coefficient of variation of the echocardiographic measurements in our laboratory was 2.5%. The following data were recorded; diameters of the left ventricular (LV) cavity at systole and diastole, the thickness of the posterior wall and the septum (both at diastole and systole), and left atrium at systole. Such data were used to calculate the relative thickness of the LV, the LV mass, and the LV mass index. After the tricuspid regurgitation was identified with a continuous-wave Doppler, systolic pulmonary arterial pressure (SPAP) was calculated using the following equation [7, 8]:

SPAP = 4 × (tricuspid systolic jet) 2 + 10 mmHg (estimated right atrial pressure). In the absence of tricuspid regurgitation, pulmonary artery pressure was calculated using acceleration time across the right ventricular outflow tract (ATRVOT) as per the formula [9]:

SPAP = 90 - 0.6 × ATRVOT in mmHg.

Pulmonary hypertension was defined as SPAP ≥35 mmHg [10]. The severity of PH was defined as: mild (SPAP 35-45 mmHg), moderate (SPAP 46-55 mmHg), and severe (SPAP> 55mmHg).

Statistical analysis

Baseline characteristics were assessed with standard descriptive statistics. The estimated glomerular filtration rate (eGFR) was calculated using the Cockcroft Gault formula. The normality of data was tested by the Kolmogorov-Smirnov test. If the data was not found to be continuous then the non-parametric test was used. Continuous variables were presented as mean ± standard deviation and median with Interquartile Range (IQR) (as applicable). Quantitative variables were compared using independent t-test (for normal distribution), z-test (when the distribution is not normal) and Mann-Whitney test (for non-parametric data) between two groups. For quantifying association, odds ratio with 95% confidence interval was used and significance of odds ratio was calculated by Fisher’s-Exact Probability statistic. The data were entered in Mocrosoft Excel (Microsoft Corporation, Redmond, WA, USA) spreadsheet, and analysis was done using an advanced statistical tool of Microsoft Excel 2010. p<0.05 was considered statistically significant.

## Results

In the present study, a total of 82 cases (71.9% men and 28.1% women) were enrolled. The mean age was 43.48±14.65 years. Out of 82 patients, 15 (18.3%) were diabetic and 50 (60.9%) were hypertensive. The majority of the patients in this study had chronic glomerulonephritis (30%), while others had diabetic kidney disease (18%), chronic interstitial nephritis (15%), or of unknown etiology (37%). In this study, PH was found in 21 (25.6%) cases with a men-to-women ratio of 2:1. Out of 21 cases of PH, mild PH was found in 7 (33.3%) cases, moderate in 14 (66.7%) and cases with severe PH were none. Non-PH group and PH group did not differ significantly when compared for mean age (p=0.39), sex (p=0.53), body mass index (p=0.74), duration of HD (p=0.55) or interdialytic weight gain (p=0.14) and the type of vascular access used for HD (p=0.11) (Table 1). The two groups did not differ significantly when compared for levels of hemoglobin, serum creatinine, eGFR, CRP, and serum albumin (Table 1). The mean level of both A1AG and pro-BNP was significantly higher in patients with PH as compared to those who were without PH (A1AG: 2920 mcg/mL vs 2250 mcg/mL, p=0.02; pro-BNP: 22231 pg/mL vs 10840 pg/mL, p=0.04) (Table 1). Echocardiographic parameters showed a significant difference between the two groups when compared for ejection fraction (60.98±6.21 v/s 57.95±5.33 p=0.005) and inferior vena cava diameter (1.72±0.25 cm v/s 1.93±0.24 cm, p=0.003) (Table 1).

**Table 1:** Characteristics of patients with ESRD undergoing maintenance hemodialysis A1AG, Alpha-1 acid glycoprotein; CRP, C-reactive protein; eGFR, estimated glomerular filtration rate; ESRD, end-stage renal disease; IVC, inferior vena cava; MHD, maintenance hemodialysis; pro-BNP, Pro-brain natriuretic peptide; PH, pulmonary hypertension.

Characteristic	ESRD on MHD Mean±SD/ Median (IQR) n=82	Pulmonary hypertension		P-value
		Absent	Present	
Age [Years]	43.48±14.65	41.10±14.33	44.29±14.78	0.39
Gender				0.53
Male	59 (71.9)	45 (73.8)	14 (66.7)	
Female	23 (28.1)	16 (26.2)	7 (33.3)	
Diabetes Mellitus	15 (18.3)	11 (18.0)	4 (19.0)	0.91
Hypertension	50 (60.9)	36 (59.0)	14 (66.7)	0.54
Arterio-venous fistula	60 (73.2)	42 (68.9)	18 (85.7)	0.11
Duration of dialysis [Months]	(15.34±11.64)	(16.67±15.20)	(14.89±10.25)	0.55
Inter-dialytic weight gain[kg]	(1.67±0.37)	(1.65±0.31)	(1.76±0.42)	0.14
Body mass index [kg/m^2^]	(20.52±2.54)	(20.46±2.44)	(20.62±2.85)	0.74
Hemoglobin [g/dL]	8.92±2.0	9.01±2.19	8.65±1.57	0.49
Creatinine [mg/dL]	6.4±2.55	6.58±2.70	5.89±2.02	0.32
eGFR [mL/min]	12.9±5.5	12.69±5.45	13.85±5.56	0.33
Albumin [g/dL]	3.64±0.65	3.68±0.65	3.53±0.67	0.39
CRP [mg/dL] (IQR)	6.1 (3.2-9.1)	5.4(3.2-8.4)	7.97(3.42-22.2)	0.15
A1AG [mcg/mL] (IQR)	2340 (1634.5-4000)	2250 (1184.5-3970)	2920 (2330-4000)	0.02
Pro BNP [pg/mL]	15550.5 (2558-35000)	10840 (1897-35000)	22231 (12356-35000)	0.04
Ejection fraction (%)	60.2±6.1	60.98±6.21	57.95±5.33	0.005
IVC Diameter (cm)	1.80±0.26	1.72±0.25	1.93±0.24	0.003

In a linear correlation, SPAP was significantly associated with A1AG (r=0.23, p=0.04) and pro- BNP (r=0.28, p=0.01) while not with ejection fraction (r=-0.21, p=0.08). On multiple regression, SPAP was significantly associated with A1AG (p=0.04) and with pro-BNP with a p-value of 0.05. Other parameters like diabetes, hypertension, duration of HD, body mass index, hemoglobin, eGFR, ejection fraction, high sensitivity CRP and albumin did not significantly affect SPAP, which were determinants for PH (Table 2).

**Table 2:** Multiple linear regression analysis with SPAP ≥35 mmHg as a dependent variable in CKD patients A1AG, alpha-1 acid glycoprotein; BMI, body mass index; CKD, chronic kidney disease; CRP, C-reactive protein; DM, diabetes mellitus; EF, ejection fraction; eGFR, estimated glomerular filtration rate; Hb, hemoglobin; HTN, hypertension; MHD, maintenance hemodialysis; pro-BNP, pro-brain natriuretic peptide; SE, standard error; SPAP, systolic pulmonary artery pressure.

Variables	Unstandardized coefficient (B)	SE	t	p-value	95% CI Lower Upper	
Constant	30.2	21.9	1.4	0.17	-13.7	74.1
eGFR	0.36	0.29	1.25	0.22	-0.22	0.94
Protein	-0.96	1.87	-0.51	0.61	-4.71	2.78
Albumin	-0.38	2.71	-0.14	0.89	-5.80	5.03
Pro-BNP	<0.001	<0.001	1.90	0.05	-0.00	0.00
CRP	-0.2	0.06	-0.34	0.73	-0.14	0.1
A1AG	<0.01	0.00	2.00	0.04	0.00	0.00
EF	-0.025	0.23	-1.10	0.27	-0.71	0.21
DM	0.47	3.79	0.12	0.90	-7.10	8.06
HTN	2.52	2.97	0.85	0.40	-3.42	8.47
Duration of MHD	0.01	0.14	0.07	0.94	-0.27	0.29
BMI	0.55	0.54	1.02	0.31	-0.52	1.62
Hb	-0.5	0.78	-0.64	0.52	-2.05	1.05

## Discussion

The pro-BNP was used as a marker of volume overload which was significantly higher in patients with PH. Furthermore, inflammation has been identified as a risk factor for atherosclerosis in these patients [6]. The patients undergoing HD are at a higher risk of developing inflammation due to factors such as exposure of blood to dialysis membranes, usage of non-sterile dialysate, cytokine retention, acidosis, and subclinical infections [11, 12]. The newer mechanisms proposed for the development of PH include the complex interaction of micro-inflammatory state and fluid overload. Further, the micro-inflammatory state is influenced by the molecular and ionic constitution of the body, which is easily altered in CKD, as shown by the influence of sodium on the micro-inflammatory state in CKD patients [13].

Human A1AG, a 41-43-kDa glycoprotein is a major acute-phase protein with inflammatory and immunomodulating properties observed in patients with CKD [14]. It consists of a single chain of 183 amino acids and 45% of its molecular weight comprises carbohydrate content that is connected in the form of five to six highly sialylated complex-type-N-linked glycans. As most acute phase proteins, its serum concentration increases in response to systemic tissue injury, inflammation or infection, and these changes in serum protein concentrations have been correlated with increases in hepatic synthesis [15]. A positive correlation between the logarithm of serum A1AG and serum creatinine levels has been observed. Docci et al. reported a significant rise in levels of serum A1AG with rising in serum creatinine level >10 mg/dL, showing the occurrence of molecule retention with a fall in renal function [16]. In our study, alpha-1-acid glycoprotein was found to be significantly higher in patients of PH indicating that inflammation played a significant role in the development of PH. However, this study did not show a significant difference between the CRP levels of the two groups. In a previous study, no significant difference in CRP was found between non-PH and PH groups [17]. However, in another study, a significant difference in CRP was found between non-PH and PH groups [18]. In the present study, it can be due to the high prevalence of infections which causes elevation of CRP in both non-PH and PH groups, thus masking the possible significant difference which was shown in previous studies.

Since the presence of arteriovenous (AV) fistula causes shunting of blood from systemic arteries to systemic veins, leading to an increased venous return to the heart, which in turn leads to increased pulmonary blood flow. Thus, AV fistula can contribute to the development of PH. Pulmonary hypertension has been shown to be associated with AV fistula by some authors [5,19] but others have shown no association [17,20]. This study too does not show any association between PH and AV fistula. In a previous study, PH was shown to be associated with duration of CKD, duration of HD, blood urea nitrogen, serum creatinine and eGFR [5] but this study does not show any association with these parameters.

Among echocardiographic parameters, ejection fraction was found to be significantly lower in patients of PH (p=0.005) indicating that LV systolic dysfunction, which also results in overloading of pulmonary vessels is associated with PH. High levels of pro-BNP are strongly associated with LV hypertrophy and dilatation and systolic dysfunction in patients with CKD [21]. These elevations have been partly explained by chronic volume overload, as observed in the PH group of this study.

## Conclusions

The present study showed higher levels of inflammatory markers A1AG and pro-BNP while lower levels of ejection fraction in patients undergoing HD indicating a significant association with PH. The incidence of PH can be prevented to some extent with effective management of fluid load in these patients. This will also reduce the micro-inflammatory state in patients undergoing HD which itself is an independent risk factor for the development of PH and other cardiovascular complications in these patients.
